# Increasing Food Expenditure in Long Day-Care by an Extra $0.50 Per Child/Day Would Improve Core Food Group Provision

**DOI:** 10.3390/nu12040968

**Published:** 2020-03-31

**Authors:** Ros Sambell, Ruth Wallace, Johnny Lo, Leesa Costello, Amanda Devine

**Affiliations:** School of Medical and Health Sciences, Edith Cowan University, Joondalup, 6027 Perth, Australia; ruth.wallace@ecu.edu.au (R.W.); j.lo@ecu.edu.au (J.L.); l.costello@ecu.edu.au (L.C.); a.devine@ecu.edu.au (A.D.)

**Keywords:** childcare, budget, long day-care, food groups, early childhood education and care, discretionary, sugar, sodium, oil equivalents

## Abstract

Early childhood education and care services are a significant feature of Australian family life, where nearly 1.4 million children attended a service in 2019. This paper reports on the cost of food provided to children in long day-care (LDC) services and extrapolates expenditure recommendations to support food provision compliance. A cross-sectional audit of LDC services in metropolitan Perth was conducted to determine food group provision by weighing raw ingredients of meal preparation—morning tea, lunch, and afternoon tea (MT, L, AT). Ingredients were costed at 2017 online metropolitan pricing from a large supermarket chain. Across participating services, 2 days of food expenditure per child/day ranged between $1.17 and $4.03 across MT, L, AT, and averaged $2.00 per child/day. Multivariable analysis suggests that an increase of $0.50 per child/day increases the odds of a LDC service meeting >50% of Australian Dietary Guideline (ADG) recommendations across ≥4 core food groups by fourfold (*p* = 0.03). Given the fact that the literature regarding food expenditure at LDC services is limited, this study provides information about food expenditure variation that impacts planning and provision of nutritionally balanced menus recommended for children. An average increase of food expenditure of $0.50 per child/day would increase food provision compliance.

## 1. Introduction

There is strong evidence that dietary intake in the first thousand days of life will impact a child’s physical and mental potential in the short, medium, and long term [1,2,3,4], and optimal nutrition has been seen as a means to prevent chronic diseases [5]. There is an increasing amount of evidence that recommends early childhood education and care (ECEC) services be an integral part of chronic disease prevention and suggests that the government should promote standards for onsite meals [6]. ECEC services are a significant feature of Australian family life. In 2019, nearly 1.4 million children attended a licensed service, either long day-care (LDC), family day-care, or out of school hours care [7]. LDC services were the most commonly utilised form of centre based care, where there was the highest attendance (58.4%) and, on average, children spent approximately 29 hours per week at these services, although nearly 6% of children typically spend more than 50 hours per week at these services [8]. LDC usually operates for 10 hours per day from Monday to Friday, provides full-time or part-time care, has an approved kindergarten program taught by a qualified teacher, and services that must comply with a range of legislative requirements [9]. Over the past two decades, there has been an increased utilisation of LDC, which is due in part to the increase in women in the labour force with young children and, more specifically, as a result of flexible work arrangements [10,11]. The LDC environment provides a significant setting where the nutritional status of children can be influenced during critical periods of growth and development [12]. Although the importance of healthy eating is well known, one in four Australian children are classified as overweight or obese [13]. The associated annual direct cost to the Australian healthcare system is estimated at $17 million and is predominantly related to hospital treatment [14]. Childhood obesity, once established, is difficult to reverse, and children who have experienced obesity are more likely to continue this trend into adulthood, increasing their risk of chronic diseases such as cardiovascular disease, type 2 diabetes, and some cancers [15]. The World Health Organization (WHO) recognises the importance of preventing these conditions and specifically highlights ECEC settings as a unique environment for community-based obesity prevention interventions [16].

The World Health Organization recommends all meals served in formal ECEC settings meet “healthy dietary guidelines” [17], compared to Australian guidelines [18], which make little reference to food provision requirements for these settings. Recent Australian findings suggest that the nutritional quality of meals provided in this setting may be sub-optimal [19,20,21,22]; discretionary foods are overprovided [23] and do not typically meet national recommendations for core food group provision [22]. Given the increasing numbers of children attending ECEC, in particular LDC services and the lack of food group compliance provided therein, food environments, including provision, should be a focus in LDC services.

There are many factors influencing the provision of food in LDC settings, including the personal characteristics of staff; relationships between children, staff, and parents; staff training; workplace characteristics; culture; and the policy and regulatory environment—the interplay of these factors may affect the quality of the food environment provided at LDC [24]. One important factor is the availability of financial resources that determine the adequacy of expenditure on food. There is evidence confirming food prices have a direct effect on diet quality, more specifically, low cost foods (typically with a high content of sugars and fats) are linked with a low quality diet [25]. Food expenditure may be influenced by socioecological determinants, such as organisational control at a broader macro system level [26]. For example, at an organisational level, the dollar value of food expenditure may be dictated by the company policy of a large LDC corporate provider, or be influenced by the individual preferences of an independent LDC owner. In either case, these macro system influences should be a consideration when addressing food expenditure, given that lower food expenditure is often associated with poorer quality diets [27].

The National Quality Standards (NQS) stipulate that food provided at LDC services should be consistent with the Australian Dietary Guidelines (ADG) [18], yet the minimum expenditure required to meet these standards is not stipulated [28]. It is acknowledged that food expenditure, regardless of adequacy, is likely to influence the nutritional quality of the food provided at LDC [24]. There is a paucity of national and international research literature pertaining to food expenditure for LDC services. Therefore, the purpose of this paper is to report the cost of food provided to children in 30 West Australian metropolitan LDC services and extrapolate expenditure recommendations to guide LDC services towards food provision compliance, when compared with the Australian Dietary Guidelines, for all core food groups.

## 2. Materials and Methods 

A cross-sectional audit of LDC services in metropolitan Perth was conducted to determine food group provision by weighing the raw ingredients used to prepare each meal: morning tea, lunch, and afternoon tea (MT, L, AT). This study was approved by the Human Research Ethics Committee (HREC), Edith Cowan University (#18486).

A minimum sample size of 27 services was required to detect a medium (Cohen’s *d* = 0.5) effect size using the Wilcoxon signed-rank test at the 5% level of significance and 80% power [29,30]. Data were collected over a 3 year period from 10 services in 2015, 9 services in 2016, and 11 services in 2017, providing a total sample of 30 services. The results reported in this paper are an outcome of a larger study, and more detailed methods have been published [31].

### 2.1. Recruitment

Inclusion criteria for service recruitment included being located in the Perth metropolitan area (postcodes between 6000–6199) in Western Australia, on site food preparation, and providing a service for >8-h per day in a working week. LDC services were randomly selected utilising a publicly available national register accessed from the Australian Children’s Education and Care Authority (ACECQA) [28]. Every 10th service was telephoned and invited to participate in the study. If services met the inclusion criteria and agreed to participate, an information letter was emailed and followed up by telephone 2 days later to schedule suitable days for data collection. Service demographics were collected, which included postcode; number of children (by age group); number of staff consuming meals on the day of data collection; and whether the service was a community or privately run service, at the centre, from the service cook and/or director. For the purpose of this comparison analysis, costing was based on food provided to 2–3-year-old children (*n* = 740). Of the services, 9 were not for profit, 1 was government-run, and 20 were privately run. Furthermore, it is important to note that staff are required to participate in meal times with children, the number of staff sharing a meal with children was captured to ensure the calculations accurately reflected the serves provided at MT, L, or AT. Services’ socio-economic status were ranked on the basis of Socio-Economic Indexes for Areas (SEIFA) ranking [32], and 12 services were ranked between 1–4, 5 of these services were ranked 1, 7 services were ranked 5, and 11 services were ranked from 6–10 (2 of these services were ranked 10).

### 2.2. Data Analysis

The raw ingredients of all foods/meals provided at participating LDC services across MT, L, and AT were weighed and itemised as described in a published protocol paper by same authors [31] and entered into Foodworks for nutrient analysis [33]. This analysis categorised ingredients according to the five core food groups, as defined by the ADG: grains (cereals); vegetables, legumes, and beans; fruits; meat and meat alternatives; and dairy and dairy alternatives [18]. In addition, sugar, sodium and discretionary foods were totalled and compared to recommendations (Table 1). The total number of serves provided across each of the five core food groups was calculated and compared to the >50% recommendation for a child aged 2–3 years for each respective food group [22] (Table 1).

In addition to the core food groups, the fat allowance was based on the ADG [18], and sugar provision was compared with the WHO guideline that diets should provide < 10% of total energy from sugar [34]. Dietary sodium was compared to the Australian upper limit of the adequate intake recommendation for children aged 1–3 years (1000 mg/day) [35]. To be consistent with the concept that at least half of a child’s dietary intake is provided whilst attending ECEC, comparison to half of these values was applied. Discretionary foods were compared to “0” serves as recommended by the Eat for Health (36)].

Discretionary foods are not represented as a food group in Foodworks [33]. As a result, discretionary foods were captured manually and the food weights were converted to a serve based on ADG recommendations, that is, one serve of discretionary food equals 600 kJ [36].

Each individual ingredient was costed using 2017 online metropolitan pricing from a large Australian supermarket chain. Store-branded products were chosen where available, no sale prices included, and products were sorted by price and the lowest priced product was selected. To increase consistency in pricing, the same product price was used consistently across a particular food. For example, all varieties of cow’s milk (full cream, Hi-Lo, skim, etc.) were entered at the same standard price.

The study sought to identify differences in food group provision among services categorised by their food expenditure using multivariate analysis of variance (MANOVA). Post-hoc ANOVA with Holm–Bonferroni-corrected *p*-values are presented for comparison within each food group.

Logistic regression modelling was used to determine whether time period for data collection, service type (private or community), socio-economic index (based on the Socio-Economic Indexes for Areas (SEIFA) [32]), and food expenditure (in the form of cost per child) had an impact on LDC being compliant with ADG recommendations for four or more core food groups (Table 1). Authors deemed, testing for compliance with four or more core food groups would be the most useful for translation into the field. All analyses were implemented in IBM SPSS version 23 [37]. Statistical significance is achieved if *p* < 0.05.

### 2.3. Exclusion Criteria

Exclusion criteria included previous participation or declined participation, or being located outside the metropolitan area of Perth.

## 3. Results

Of the 30 LDC services that completed the 2 day weighed ingredient food provision intake study [31], 20 (66.7%) were privately-owned.

### Food Expenditure vs. Food Group Provision

**Figure 1:**
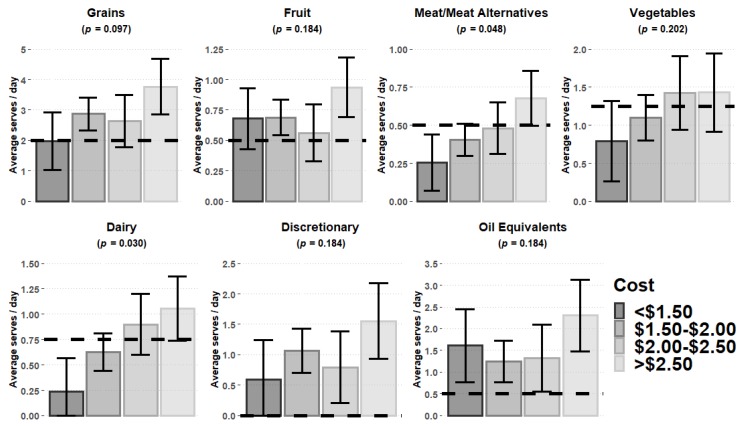
Cost ($) per child versus average serves/day of each core food group, discretionary foods, and oil equivalents. Dotted line equals recommended serves for each category (50%). Error bars represent the ±95% confidence interval. Discretionary foods have been based on a recommendation of “0” serves. Oil equivalents based on Australian Dietary Guidelines (ADG) recommendations for a child aged 2–3 years [18].

Food expenditure per child/day ranged between $1.17–$4.03 across MT, L, and AT, and averaged $2.00 per child/day (Figure 1). The food expenditure of two thirds of participating services (*n* = 20; 66.7%) was between $1.50–2.50 per child/day, although five services spent less than $1.50, and five services spent more than $2.50 per child/day.

Using MANOVA, significant differences in food group provision between the lowest and highest daily expenditure were identified in relation to the food expenditure calculated (*p* = 0.014). Figure 1 presents a summary of the post-hoc comparisons for the core food groups, discretionary foods, and oil equivalents as total contribution to cost across the three meal opportunities, and shows significant reduction in the provision of meat and meat alternatives (*p* = 0.048) and dairy (*p* = 0.030), core food groups with reduced food expenditures. On average, services with food expenditures less than $2.00 ± 0.56 per child/day (median ± interquartile range is 1.92 ± 0.53) did not meet the 50% recommended level for dairy and meat and meat alternatives groups. In our sample, the mean serves were 0.24 and 0.25 serves, respectively, for dairy and meat/meat alternatives. 

**Figure 2:**
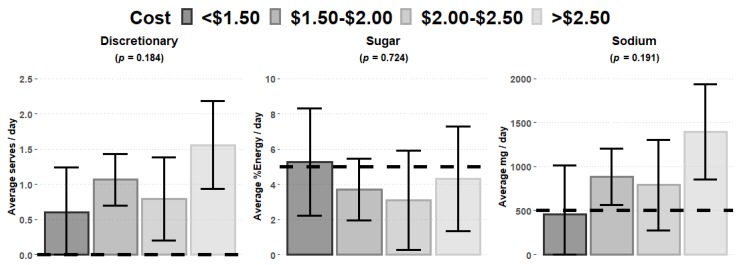
Cost relationship with discretionary food, sugar, and sodium provision. Dotted line equals recommended serves for each core food group (50% ADG). Error bars represent the ±95% confidence interval. Sugar comparator is 50% of 10% of total energy [38]. Sodium comparator is 50% upper limit of Nutrient Reference Value for child aged 1–3 years (1000 mg/day) [35].

Figure 2 highlights there was no significant association for discretionary food, sugar, and sodium with food expenditure. However, it is worth noting that discretionary foods and sodium were overprovided when compared with recommendations.

Univariable and multivariable logistic regression analyses revealed that the year of data collection, service type (private or community-based), and socio-economic index did not have any significant influence on whether a participating service was compliant with ADG recommendations for four or more core food groups. The level of food expenditure was the only variable that had a significant influence on core food group compliance. The multivariable model suggests that an increase of $0.50 per child/day increases the odds of an LDC service meeting >50% of ADG recommendations across four or more core food groups by fourfold (*p* = 0.03) as per Table 2. For example, all five services that spent less than $1.50 per child/day were non-compliant with 50% of the recommended serves for at least three of the five core food groups. In contrast, of the five services with food expenditure greater than $2.50, three were compliant for all five core food groups, and one service was compliant in three core food groups, whereas the fifth one was only compliant in two (grains (cereals) and fruit).

## 4. Discussion 

The purpose of this paper was to report the cost of current food provision in 30 West Australian metropolitan LDC services and make food expenditure recommendations to facilitate compliance with more than 50% of the ADG for morning tea (MT), lunch (L), and afternoon tea (AT) across all core food groups.

The average food expenditure per child/day was calculated at $2.00, but varied widely, ranging between $1.17 and $4.03 per child/day. Lower food expenditures were associated with a lower likelihood of participating services meeting the ADG core food group recommendations for children (Figure 1). LDC services with a food expenditure <$1.50 per child/day did not meet the recommendations for three core food groups: meat and meat alternatives, vegetables, or dairy and dairy alternatives (Figure 1). On the basis of the mean serves of 0.24 and 0.25 serves calculated for dairy and meat/meat alternatives, respectively, services would need to increase their provision of these two core food groups by three- and twofold, respectively, to meet the 50% ADG recommendations. Although discretionary foods, sodium and oil equivalents did not show any statistical association between level of expenditure, these were overprovided regardless of expenditure levels. As a result, the overall daily dietary intake of children at LDC may be nutritionally sub-optimal, increasing the risk of obesity, non-communicable diseases, impaired growth and development, and nutrient deficiencies [39,40]. 

The practical implications of these findings are complex and required further modelling to direct LDC services to translate information into practice. Cost modelling demonstrated an increase in food expenditure of $0.50 per child/day would increase compliance with ADG recommendations for four or more core food groups. Coupled with evidence that meat and meat alternatives and dairy would benefit most from this increased expenditure, recommendations for allocation of this expenditure to increase core food group compliance are suggested (Table 3). 

Table 3 demonstrates the most cost-effective additions to a daily LDC menu to increase provision for meat and meat alternatives and dairy, as well as to improve compliance to core food group recommendations, that is, one serve of cooked legumes and one serve of milk at MT, L, AT. It is important that LDC menus offer a variety of foods, and thus it is likely that LDC service staff will need more support to optimise the nutritional content of their menus whilst incorporating budget considerations/limitations.

This research shows a significant overprovision of discretionary foods at LDC in contrast to ADG recommendations. It is well known that among the Australian population dietary expenditure is changing and a greater proportion of household expenditure is allocated to discretionary foods [41,42]; this is likely to be replicated in LDC menus. This may, in part, be explained by the lack of nutrition education requirements for staff who prepare foods for LDC [43], the low cost and accessibility [44] of discretionary foods, and the ever increasing “normalised” consumption of such foods [41]. Therefore, services should replace discretionary foods on their menu with foods from the core food groups, particularly, meat and meat alternatives, dairy, and vegetables, moving menus closer to core food group compliance, and ultimately optimising nutrition for children. 

Menu change may require a shift in organisational culture to facilitate an attitudinal shift regarding the perceived cost of healthy versus discretionary foods and subsequent policy inclusions to support this. 

Conversely, regardless of the level of the daily food expenditure, all participating LDC services exceeded the recommendations for two core food groups: grains (cereals) and fruit (Figure 1). Carbohydrate foods such as pasta and rice can be cheap and satiating but may be overprovided because of their low cost. Overprovision of fruit, refined carbohydrates, and discretionary foods could be displacing the opportunity for provision of other core food groups, which may increase the risk of nutritional deficiencies and reduce exposure to a variety of foods [45].

Research has also indicated that LDC services are not achieving core food group provision recommendations [20,22,46], and the range and distribution of food expenditure, evident from this study, could explain this inadequate core food group provision that may contribute to a nutritionally sub-optimal food intake for children attending LDC. Evidence from this study suggests that services who spend less on food are less likely to be compliant with ADG recommendations for core food groups. These findings are supported, in part, by a qualitative U.S. study of LDC providers (*n* = 16), which reported some, but not all, participants believing that limited expenditures impacted their ability to provide healthy meals [47], and a Polish study of preschools that suggested the level of food budget would likely impact purchasing capacity, particularly more expensive food products [48]. A further study conducted by Ottenetal [49] claimed that restrictive food budgets would likely have a negative impact on food decisions, on the basis of how services or individuals interpret regulations and how personal values can affect food purchasing decisions.

This study is not without limitations. The pricing of meal ingredients was based on foods available in a metropolitan area, and it is quite likely that rural and remote pricing would be higher. Increasing the number of days that data were collected would provide a more accurate representation of costings. Services were aware that ingredient measurement was going to be undertaken and their choice of products may have changed due to social desirability bias, thus affecting the costing. Despite data being collected over 3 years, costing was based on the most recent year of data collection and dependent on price variation of food, and this may have under- or overestimated costings. Additionally, the influence of pricing would be affected by seasonal variation, and this was not captured in the costing estimates because this was undertaken at one time point.

## 5. Conclusions

These data demonstrated significant variation in LDC food expenditure, and these variations impact the provision of nutritionally balanced menus recommended for children attending LDC services. The results of this study suggest an increase in average food expenditure of $0.50 per child/day would significantly increase core food provision compliance.

The type of foods offered to children, how they are offered, and how and when they are consumed, is a significant public health issue due to the impact this has on children’s future food decisions. [42,50,51,52,53]. This is especially true for children attending LDC, primarily because attendance rates are increasing. The setting itself provides an opportune context in which to combat obesity, as well as to optimise growth and development. Further, the LDC environment is ideally positioned to support the establishment of healthy food preferences and habits because it takes place in such a critical period of influence in the child’s life, influencing food (and nutrient) intake as well as shaping future preferences. In recognizing that research regarding food expenditure at LDC services is limited, our research establishes new knowledge by demonstrating that the level of food expenditure does reflect the subsequent nutritional value of the food provided and likely the compliance to dietary recommendations.

It is essential that children receive nutritionally appropriate food whilst attending LDC services to reduce obesity risks and support long-term health and wellbeing. Addressing appropriate and sufficient levels of food expenditure available to LDC staff could provide a mechanism to improve core food group provision compliance and provide young Australian children with the best possible start to life.

There is no jurisdictional legislation or guideline that stipulates appropriate levels of food expenditure in the Australian ECEC sector. It is hoped that our study will espouse the drafting of legislation and policies by demonstrating that variation in expenditure does impact on food group provision. Children and staff would ultimately benefit from the relevant legislative and regulatory bodies supporting menu development that recommends a level of expenditure sufficient to achieve adequate provision of core food groups. This policy-based action could be further supported by training LDC staff on topics such as providing healthy meals on a limited budget, improving menu planning skills, and increasing staff confidence to address food budget issues. Further research on the impact of changes to legislature, policy, and access to training to support financial resourcing for adequate food provision are critical.

## Figures and Tables

**Table 1:** Recommended daily core food group serves for a child aged 2–3 years old and serves per >50% provision. Additional comparison for oil equivalents, added sugars, sodium, and discretionary foods.

Food Group [18]	ADG Recommendation [18]	>50% Serves
Vegetables, legumes, beans	2.5	1.25
Fruit	1	0.5
Grains (cereals)	4	2
Meat and meat alternatives	1	0.5
Dairy and dairy alternatives	1.5	0.75
**Additional comparisons**		
Unsaturated fats/oil equivalents	6.5 g (as a midpoint between ADG recommendations for 2–3 and 3–12 years old)	3.25 g
Added sugars	10% of total energy	50% of 10% of total energy
Sodium	1000 mg/day	500 mg/day
Discretionary foods	0 serves	0 serves

**Table 2:** Logistic regression modelling of core food group recommendation compliance against service demographics and food expenditure **.

Variable	Response	*n* (%)	Univariable		Multivariable	
			OR (95% CI)	*p*-Value	OR (95% CI)	*p*-Value
**Year**	**Overall**			0.602		0.407
	2015	10 (33.3%)	1.00 (Ref)		1.00 (Ref)	
	2016	11 (36.7%)	3.4 (0.3, 40.9)	0.330	0.63 (0.04, 9.00)	0.737
	2017	9 (30%)	3.0 (0.3, 35.3)	0.383	0.05 (0.001, 4.353)	0.190
Service	Private	20 (66.7%)	1.00 (Ref)		1.00 (Ref)	
	Community-based	10 (33.3%)	1.7 (0.3, 9.8)	0.544	2.3 (0.2, 25.5)	0.505
SEIFA	Range 1–10		1.2 (0.4, 3.5)	0.794	1.1 (0.7, 1.8)	0.658
Cost per child (per 50¢ increment)	Range $1.17–$4.03	2.00 ± 0.59	2.6 (1, 6.7)	0.050 *	4.0 (1.1, 13.9)	0.030 *

* Significant at the 5% level; OR = odds ratio; CI = confidence interval; 1.00 (Ref) = reference level; ** 2017 pricing.

**Table 3:** $0.50 expenditure recommendations for meat and meat alternatives and dairy to improve compliance with four or more core food groups for >50% provision of ADG per child.

Meat and Meat Alternatives Food Group	Cost	Serve Size	Dairy Food Group	Cost	Serve Size
Lean beef mince—raw ($1.30/100 g)	$0.50/39 g	0.5	Milk—reduced fat ($0.10/100 ml)	$0.50/500 mL	2.0
Lean chicken—raw ($0.90/100 g)	$0.45/50 g	0.5	Hard cheese ($1.20/100 g)	$0.50/42 g	1.0
Canned legumes—cooked ($0.19/100 g)	$0.46/240 g	2.0	Yoghurt ($0.35/100 g)	$0.50/142 g	0.7

## References

[1] Spence A.C., Campbell K.J., Lioret S., McNaughton S.A. (2018). Early childhood vegetable, fruit, and discretionary food intakes do not meet dietary guidelines, but do show socioeconomic differences and tracking over time. J. Acad. Nutr. Diet..

[2] Nyaradi A., Li J., Foster J., Hickling S., Jacques A., O’Sullivan T.A., Oddy W.H. (2015). Good-quality diet in the early years may have a positive effect on academic achievement. Acta Paediatr..

[3] Nyaradi A., Foster J.K., Hickling S., Li J., Ambrosini G.L., Jacques A., Oddy W.H. (2014). Prospective associations between dietary patterns and cognitive performance during adolescence. J. Child Psychol. Psychiatry.

[4] Nyaradi A., Li J., Hickling S., Foster J., Oddy W.H. (2013). The role of nutrition in children’s neurocognitive development, from pregnancy through childhood. Front. Hum. Neurosci..

[5] Australian Institute of Health and Welfare (2018). Nutrition Across the Life Stages.

[6] Mozaffarian D., Angell S.Y., Lang T., Rivera J.A. (2018). Role of government policy in nutrition—Barriers to and opportunities for healthier eating. BMJ.

[7] Department of Education and Training, Department of Education and Training (2019). Early Childhood and Child Care in Summary: Sept Quarter 2019.

[8] Department of Education and Training, Department of Education and Training (2018). Early Childhood and Child Care in Summary: June Quarter 2018.

[9] Queensland Government (2019). Long Day Care, Queensland, Australia: Early Childhood Information Service. https://www.qld.gov.au/families/babies/childcare/types/long.

[10] ABS (2010). ABoS. Child Care. https://www.abs.gov.au/AUSSTATS/abs@.nsf/Lookup/4102.0Main+Features50Jun+2010.

[11] ABS (2017). ABoS. Child Care. https://www.abs.gov.au/ausstats/abs@.nsf/Latestproducts/4402.0Media%20Release1June%202017?opendocument&tabname=Summary&prodno=4402.0&issue=June%202017&num=&view=.

[12] Briley M., McAllaster M. (2011). Nutrition and the child-care setting. J. Am. Diet. Assoc..

[13] Australian Institute of Health Welfare (2019). Overweight and Obesity: An Interactive Insight.

[14] Brown V., Moodie M., Baur L., Wen L., Hayes A. (2017). The high cost of obesity in Australian pre-schoolers. Aust. N. Z. J. Public Health.

[15] Kelsey M.M., Zaepfel A., Bjornstad P., Nadeau K.J. (2014). Age-related consequences of childhood obesity. Gerontology.

[16] World Health Organization [WHO] (2012). Population-Based Approaches to Childhood Obesity Prevention.

[17] Production W.D., World Health Organisation (2016). Report of the Commission on Ending Childhood Obesity.

[18] National Health and Medical Research Council (2013). Australian Dietary Guidelines.

[19] Bell L., Hendrie G., Hartley J., Golley R. (2015). Impact of a nutrition award scheme on the food and nutrient intakes of 2- to 4-year-olds attending long day care. Public Health Nutr..

[20] O’Halloran S.A., Lacy K.E., Grimes C.A., Campbell K.J., Nowson C.A. (2018). Sodium content of lunches and snacks provided in australian long day care centres: A cross-sectional study. Nutrients.

[21] Finch M., Wolfenden L., Falkiner M., Edenden D., Pond N., Hardy L.L., Milat A.J., Wiggers J. (2012). Impact of a population based intervention to increase the adoption of multiple physical activity practices in centre based childcare services: A quasi experimental, effectiveness study. Int. J. Behav. Nutr. Phys. Act..

[22] Sambell R., Devine A., Lo J. (2014). Does the food group provision in early years’ education and care settings in metropolitan Perth, Western Australia, meet national dietary requirements; and how can home economics support this?. J. Home Econ. Inst. Aust..

[23] Wallace R., Costello L., Devine A. (2017). Over-provision of discretionary foods at childcare dilutes the nutritional quality of diets for children. Aust. N. Z. J. Public Health.

[24] Hirsch T., Lim C., Otten J. What’s for lunch? A socio-ecological approach to childcare nutrition. Proceedings of the DIS ‘16: Proceedings of the 2016 ACM Conference on Designing Interactive Systems.

[25] Darmon N., Drewnowski A. (2015). Contribution of food prices and diet cost to socioeconomic disparities in diet quality and health: A systematic review and analysis. Nutr. Rev..

[26] Bronfenbrenner U. (1986). Ecology of the family as a context for human development: Research perspectives. Dev. Psychol..

[27] Drewnowski A. (2015). Nutrition economics: How to eat better for less. J. Nutr. Sci. Vitaminol..

[28] Australian Children’s Education & Care Quality Authority [ACECQA] (2018). Guide to National Quality Framework. https://www.acecqa.gov.au/sites/default/files/2018-03/Guide-to-the-NQF_0.pdf.

[29] Lakens D. (2013). Calculating and reporting effect sizes to facilitate cumulative science: A practical primer for t-tests and ANOVAs. Front. Psychol..

[30] Cohen J. (1988). Statistical Power Analyses for the Behavioral Sciences.

[31] Sambell R., Wallace R., Costello L., Lo J., Devine A. (2019). Measuring food provision in Western Australian long day care (LDC) services: A weighed food record method/protocol at a service level. Nutr. J..

[32] Australian Bureau of Statistics [ABS] (2016). Socio-Economic Indexes for Areas: ABS. https://www.abs.gov.au/websitedbs/censushome.nsf/home/seifa.

[33] Xyris Software (2018). FoodWorks 9 Professional.

[34] World Health Organisation (2015). Healthy Diet. Fact Sheet No 394.

[35] National Health and Medical Research Council (2017). Nutrient Reference Values for Australia and New Zealand Including Recommended Dietary Intakes.

[36] Eat for Health (2017). Discretionary Food and Drink Choices, Australian Government—National Health and Medical Research Council. https://www.eatforhealth.gov.au/food-essentials/discretionary-food-and-drink-choices.

[37] IBM Corp. (2018). IBM SPSS Statistics for Windows.

[38] World Health Organization (2015). Guideline: Sugars Intake for Adults and Children.

[39] Hodder R.K., Stacey F.G., O’Brien K.M., Wyse R.J., Clinton-McHarg T., Tzelepis F., Nathan N.K., James E.L., Bartlem K.M., Sutherland R. (2018). Interventions for increasing fruit and vegetable consumption in children aged five years and under. Cochrane database Syst. Rev..

[40] Mennella J.A., Reiter A.R., Daniels L.M. (2016). Vegetable and fruit acceptance during infancy: Impact of ontogeny, genetics, and early experiences. Adv. Nutr..

[41] Lee A.J., Kane S., Ramsey R., Good E., Dick M. (2016). Testing the price and affordability of healthy and current (unhealthy) diets and the potential impacts of policy change in Australia. BMC Public Health.

[42] Fong M., Li A., Hill A.J., Cunich M., Skilton M.R., Madigan C.D., Caterson I.D. (2019). Modelling the association between core and discretionary energy intake in adults with and without obesity. Nutrients.

[43] Expect A Star (2018). Cook Qualification Requirements. https://expectastar.com.au/cook-qualifications-and-requirements.

[44] Australian Academy of Science (2017). Rethinking food and nutrition science: The food environment. 2017 Theo Murphy High Flyers Think Tank Discussion Paper.

[45] Wallace R. (2016). Supporting Nutrition for Australian Childcare (SNAC): The Development, Implementation and Evaluation of an Online Nutrition Education Intervention. Ph.D. Thesis.

[46] Yoong L., Skelton E., Jones J., Wolfenden L. (2014). Do childcare services provide foods in line with the 2013 Australian dietary guidelines? A cross-sectional study. Aust. N. Z. J. Public Health.

[47] Lynch M., Batal M. (2011). Factors influencing childcare providers’ food and mealtime decisions: An ecological approach. Child Care Pract..

[48] Myszkowska-Ryciak J., Harton A. (2019). Eating healthy, growing healthy: Outcome evaluation of the nutrition education program optimizing the nutritional value of preschool menus, Poland. Nutrients.

[49] Otten J.J., Hirsch T., Lim C. (2017). Factors influencing the food purchases of early care and education providers. J. Acad. Nutr. Diet..

[50] Driessen C.E., Cameron A.J., Thornton L.E., Lai S.K., Barnett L.M. (2014). Effect of changes to the school food environment on eating behaviours and/or body weight in children: A systematic review. Obes. Rev..

[51] Birch L.L., Doub A.E. (2014). Learning to eat: Birth to age 2 y. Am. J. Clin. Nutr..

[52] Cruwys T., Bevelander K.E., Hermans R.C.J. (2015). Social modeling of eating: A review of when and why social influence affects food intake and choice. Appetite.

[53] French S.A., Epstein L.H., Jeffery R.W., Blundell J.E., Wardle J. (2012). Eating behavior dimensions. Associations with energy intake and body weight. A review. Appetite.

